# Beyond Fertility: Ethical and Psychosocial Dimensions of Fertility Preservation in Children and Adolescents

**DOI:** 10.7759/cureus.117913

**Published:** 2026-10-14

**Authors:** Mira S Aridas, Kathryn Grive

**Affiliations:** 1 Medicine, University of Melbourne, Melbourne, AUS; 2 Obstetrics and Gynecology, Brown University, Providence, USA

**Keywords:** decisional regret, fertility preservation, oncofertility, ovarian tissue cryopreservation, pediatric cancer, psychosocial support, testicular tissue cryopreservation

## Abstract

This narrative review examines fertility preservation in children and adolescents receiving gonadotoxic treatment for cancer and serious non-malignant conditions, including chemotherapy, radiotherapy, and hematopoietic stem cell transplantation. It considers the impact of these therapies on future reproductive potential and summarizes current fertility preservation strategies according to sex and pubertal status. Established options for postpubertal adolescents are discussed alongside gonadal tissue cryopreservation approaches for prepubertal children and emerging experimental strategies, including insights from animal studies and the uncertainties surrounding the future clinical use of preserved tissue. Particular attention is given to the ethical complexities of fertility preservation in childhood, including informed consent, assent, parental decision-making, evolving autonomy, and procedures with uncertain future benefit. The psychological impact on children, adolescents, and their families is also considered, together with the role of multidisciplinary teams in providing timely, equitable, developmentally appropriate counseling and ongoing psychosocial support. The review concludes that fertility preservation should be regarded not as a single decision made before gonadotoxic treatment, but as a longitudinal process extending from diagnosis through survivorship and into adulthood.

## Introduction and background

Advances in pediatric medicine have significantly improved the prognosis of childhood cancers and various chronic medical conditions. Survival rates for pediatric malignancies now exceed 80%, resulting in a growing population of childhood, adolescent, and young adult survivors [1,2]. Comparable advancements have been reported in non-malignant conditions that require intensive or gonadotoxic therapies, such as hematologic disorders, rheumatologic diseases, genetic syndromes, and patients undergoing hematopoietic stem cell transplantation (HSCT) [1-3]. As survival rates increase, the focus has shifted from achieving cure alone to addressing long-term health and quality-of-life outcomes, with fertility becoming a central concern [2].

Treatment of childhood cancers, including leukemias, lymphomas, central nervous system tumors, and solid tumors, often involves multimodal regimens such as chemotherapy, radiotherapy, surgery, or transplantation. Many of these interventions are inherently gonadotoxic. Alkylating agents, platinum-based chemotherapies, and radiotherapy targeting the gonads or hypothalamic-pituitary axis can cause permanent gonadal damage, pubertal disruption, and infertility [1,3]. Notably, fertility impairment is not limited to oncology; children receiving gonadotoxic therapies for chronic non-malignant conditions or systemic diseases may also experience long-term reproductive consequences [1-3].

Growing awareness of infertility as a downstream effect of pediatric disease has prompted the development of fertility preservation (FP) strategies specifically tailored to children and adolescents. Established methods, including sperm and oocyte cryopreservation, are accessible to post-pubertal patients, while techniques such as testicular and ovarian tissue cryopreservation offer potential future fertility options for pre-pubertal children who lack other viable alternatives [2,4]. Sperm cryopreservation involves freezing a semen sample for later use in assisted reproduction, while oocyte cryopreservation involves hormonal stimulation of the ovaries followed by retrieval and freezing of mature eggs. Because prepubertal children do not yet produce mature gametes, the options available to them are surgical. In ovarian tissue cryopreservation, a portion of the ovarian cortex containing immature follicles is removed and frozen for later reimplantation. In testicular tissue cryopreservation, a small testicular biopsy containing spermatogonial stem cells, the precursor cells from which sperm develop, is frozen for potential future use. Advances in reproductive technology and oncofertility research further support the integration of fertility counseling and preservation into standard pediatric care before the initiation of gonadotoxic therapy [5,6].

Despite these developments, FP in pediatric patients presents unique psychological, ethical, and practical challenges that distinguish it from adult practice. Adults generally consent for themselves and can weigh preservation against their own reproductive intentions, whereas in pediatric care, decision-making frequently occurs during periods of acute illness and emotional distress, with limited time for deliberation. Children and adolescents may have reduced capacity to understand future reproductive implications, while parents or caregivers are forced to balance immediate survival needs with uncertain long-term reproductive outcomes. Ethical considerations related to assent, consent, experimental procedures, future autonomy, and potential decisional regret are particularly significant in this context [7-9]. In practice, clinicians and families must decide how to balance parental consent with the child’s own assent, how to respect the future reproductive autonomy of a person who cannot yet express clear preferences, and whether invasive or experimental procedures of uncertain benefit are justified. Further complexities arise in specific populations, such as children with disorders of sex development, chromosomal conditions like Klinefelter syndrome, and transgender or gender-diverse adolescents, where fertility counseling would need to account for gender identity, psychosocial well-being, and evolving personal values [10,11].

These dilemmas are most acute in prepubertal children. Although gonadal tissue cryopreservation is feasible and increasingly offered, there are currently no clinically established methods to reliably mature preserved immature germ cells into functional gametes for use in assisted reproduction [4,12]. This limitation further intensifies the psychological burden on families and raises ethical concerns regarding consent, evolving autonomy, and uncertain benefit.

Fertility discussions also carry a psychological burden that is distinctive to childhood. For adolescents, they arise alongside developing sexuality, body image, and identity, while parents may experience guilt, anxiety, and fear of making a decision their child would not have chosen. Because the consequences of these decisions may not become apparent for years or decades, their psychological impact extends well into survivorship.

Considering these clinical, ethical, and psychosocial factors, a multidisciplinary approach to FP is essential. Early, patient-centered counseling that integrates medical risk assessment, developmental stage, family values, and patient preferences is increasingly recognized as a standard of high-quality pediatric care. Nevertheless, substantial gaps persist in awareness, access, consistency of practice, and evidence-based guidance across diverse disease groups and clinical settings [5,13].

This review has three objectives: first, to summarize the fertility risks associated with gonadotoxic therapies in childhood and current FP options according to sex and pubertal status; second, to examine the ethical dilemmas specific to pediatric FP, including consent, assent, future autonomy, and the use of experimental procedures; and third, to explore the psychological impact of these decisions on children, adolescents, and their families, together with the role of healthcare providers in supporting them. By integrating clinical, ethical, and psychosocial perspectives, the review aims to support equitable, patient-centered FP care for children and adolescents at risk of infertility.

## Review

This narrative review was conducted and reported in accordance with the Scale for the Assessment of Narrative Review Articles (SANRA) [14]. A literature search was performed in PubMed to identify articles published through October 2025 that address FP in pediatric and adolescent populations and its ethical and psychological implications for patients and families, including original studies, reviews, guidelines, case reports, and trials. The search combined terms relating to FP (“fertility preservation”, “oncofertility”, “ovarian tissue cryopreservation”, “testicular tissue cryopreservation”, “sperm banking”, “oocyte cryopreservation”), pediatric populations (“child”, “adolescent”, “pediatric”, “prepubertal”), and ethical and psychosocial aspects (“ethics”, “informed consent”, “assent”, “decision making”, “decisional regret”, “psychological”, “psychosocial”, “counseling”, “parent”), using Medical Subject Headings and the Boolean operators AND and OR.

Articles were included if they addressed fertility risk, FP techniques, or the ethical or psychosocial aspects of FP in children, adolescents, or young adults, or in their families or healthcare providers. Articles focusing exclusively on adult populations and conference abstracts without full text were excluded. The search identified 1,335 records. Titles were screened for relevance, and more than 200 abstracts and full-text articles were reviewed in detail, of which 23 were selected for inclusion. Additional relevant publications, including international clinical practice guidelines and preclinical studies of experimental FP techniques, were identified through targeted searches and by reviewing the reference lists of included articles.

Information on study design, population, the FP approach, and ethical, psychological, or clinical outcomes was extracted from each included article. Given the heterogeneity of study designs, populations, and outcome measures, a formal risk-of-bias assessment and quantitative synthesis, such as meta-analyses, were not undertaken. Evidence was instead synthesized narratively and organized thematically into clinical, ethical, and psychosocial domains. For psychosocial outcomes, including decisional regret, fertility-related concerns, and experiences of counseling, findings were grouped by outcome and by population (patients, parents, and clinicians), and areas of agreement and divergence between studies were described. Where studies reported quantitative results, such as sample sizes, proportions, or scores on validated instruments, these are presented descriptively as reported in the original publications, and no data were pooled.

Current practice

As mortality in pediatric and adolescent populations continues to improve, FP has evolved from a secondary consideration to a core element of comprehensive, patient-centered care. Although oncology remains the most common indication for an FP referral, there is increasing recognition of fertility risk in hematological disorders requiring HSCT, autoimmune and inflammatory diseases treated with alkylating agents, and gender-affirming care involving gonad-suppressive medical or surgical interventions [1-3,10]. Across these diverse clinical contexts, infertility risk arises from shared mechanisms of gonadal injury related to chemotherapy, radiotherapy, surgery, or prolonged medical therapy, often delivered at a time when reproductive potential cannot yet be reliably assessed [1-3].

The magnitude of infertility risk is determined by treatment modality, cumulative exposure to gonadotoxic agents, radiation field and dose, and individual susceptibility. Alkylating agents, craniospinal or pelvic irradiation, and HSCT conditioning regimens confer the highest risk, with consequences ranging from impaired pubertal progression to premature gonadal insufficiency and permanent infertility [1,3,4,15]. Importantly, gonadal damage in children is frequently subclinical at the time of therapy, with reproductive consequences manifesting years later [2], reinforcing the need for proactive, pre-treatment fertility counseling.

In postpubertal adolescents, sperm cryopreservation and oocyte cryopreservation remain established, effective options with well-characterized safety profiles and are recommended by current international guidelines [16-18]. However, real-world uptake remains inconsistent. Institutional and qualitative studies demonstrate that despite guideline recommendations, fertility counseling and sperm banking are frequently delayed or omitted due to acute illness, time constraints, discomfort discussing sexual health, logistical barriers, and cost [15,19]. Notably, both clinicians and survivors report regret when FP opportunities are missed, highlighting the ethical imperative for systematic counseling [7].

Female pediatric patients

In postpubertal females, gonadotropin-releasing hormone analogues (GnRHa), such as triptorelin, have been explored as adjunctive strategies to mitigate chemotherapy-induced ovarian damage. Observational data suggest that GnRHa administration during chemotherapy may preserve ovarian function in patients not undergoing HSCT, but protection is incomplete and ineffective in the context of highly gonadotoxic conditioning regimens [20]. As such, GnRHa should be viewed as a potential supportive measure rather than a substitute for established FP techniques [18].

For prepubertal girls, who lack mature oocytes and cannot undergo ovarian stimulation, ovarian tissue cryopreservation (OTC) is the only FP option available before gonadotoxic therapy. In 2019, the American Society for Reproductive Medicine removed the experimental designation from OTC [21], and international guidelines now support offering it to prepubertal girls at high risk of infertility [16,18]. However, outcome data specific to prepubertal patients remain limited, and the first live birth from ovarian tissue cryopreserved before menarche was not reported until 2015 [22]. Although restoration of endocrine function and pubertal induction following reimplantation have been documented, reproductive success cannot be reliably predicted at the time of tissue collection, and practice patterns and barriers to access continue to vary between centers [23].

An additional concern is tissue quality at the time of cryopreservation. Prior exposure to chemotherapy, particularly alkylating agents, can significantly reduce follicle density and increase follicular atresia within cryopreserved ovarian tissue, even when cumulative doses fall below traditional thresholds for FP referral [24]. Animal studies help explain this vulnerability: in mice, cyclophosphamide drives dormant primordial follicles into growth, resulting in follicle loss and a “burnout” of the ovarian reserve [25], and exposure during the prepubertal period produces long-term loss of primordial follicles and impaired embryo development [26]. This raises important questions regarding the optimal timing of OTC, especially in patients who require urgent treatment or who become eligible for FP only after relapse or treatment escalation.

Risk stratification is further complicated by heterogeneity in gonadotoxic exposure. Patients with central nervous system tumors or those undergoing HSCT conditioning may face substantial infertility risk due to craniospinal irradiation, hypothalamic-pituitary axis disruption, or high cumulative alkylator exposure, even in the absence of direct ovarian irradiation [1,24]. Biomarkers such as anti-Müllerian hormone (AMH) also have limited clinical utility in this population. Prospective data in adolescent cancer patients demonstrate a uniform decline in AMH during chemotherapy, with partial recovery in a small subset after treatment, but short-term measurements do not reliably predict long-term fertility or reproductive lifespan [27,28].

FP in prepubertal girls is therefore characterized by uncertainty: an invasive procedure is undertaken in early childhood, while its benefit may not be known for decades. This makes FP in this group a values-based decision rather than solely a technical intervention, as discussed further below [7,8].

Male pediatric patients

For prepubertal boys, testicular tissue cryopreservation offers the only potential avenue for FP. While surgical retrieval has been shown to be safe with minimal morbidity, there is currently no clinically available method to mature preserved spermatogonial stem cells into functional sperm [4,12]. Families are therefore asked to consent to an invasive procedure with uncertain future utility [8].

Animal studies are relevant to this discussion because they define what families can realistically be told about the future use of stored tissue, which is central to informed consent. In non-human primates, autologous grafting of cryopreserved prepubertal testicular tissue has produced functional sperm and a live birth [29], and spermatogonial stem cell transplantation has restored spermatogenesis in macaques rendered infertile by alkylating chemotherapy [30]. In mice, complete spermatogenesis has been achieved in vitro from cryopreserved neonatal testicular tissue [31]. Until these techniques are shown to be safe and effective in humans, uncertainty regarding the ultimate reproductive potential of preserved tissue remains.

Collectively, the uncertain long-term efficacy of FP in prepubertal patients, time-critical decision-making before life-saving therapy, and substantial logistical and psychosocial barriers amplify the ethical and psychological burden faced by patients, families, and clinicians. This reinforces the need for multidisciplinary, developmentally sensitive counseling and transparent communication about both the possibilities and limitations of current FP strategies [5,6,8]. Table 1 summarizes established and experimental FP options, their main limitations, and the ethical concerns associated with each.

**Table 1:** Established and experimental fertility preservation options in children and adolescents

Option	Eligible population	Clinical status	Main limitations	Key ethical concerns
Sperm cryopreservation	Postpubertal males	Established; recommended by international guidelines [16-18]	Requires production of a semen sample; uptake limited by acute illness, time constraints, and cost [15,19]	Discussing sexual health with adolescents; privacy and confidentiality
Oocyte cryopreservation	Postmenarchal females	Established [16,18]	Requires hormonal ovarian stimulation and oocyte retrieval; may delay treatment	Invasiveness relative to age; decision-making under time pressure
GnRHa	Postpubertal females	Adjunct only; not a substitute for established methods [18,20]	Incomplete protection; ineffective with highly gonadotoxic conditioning [20]	Risk of false reassurance
Ovarian tissue cryopreservation	Prepubertal and postpubertal females	No longer considered experimental; limited outcome data before menarche [21-23]	Surgical procedure; tissue quality reduced by prior alkylating exposure [24]; reproductive success unpredictable	Proxy consent for surgery of uncertain benefit; future decisions about stored tissue
Testicular tissue cryopreservation	Prepubertal males	Experimental [4,12]	No clinically established method to use stored tissue [4,12]	Proxy consent for an invasive procedure with uncertain benefit; disposition of stored tissue
Spermatogonial stem cell transplantation and in vitro spermatogenesis	Future use of stored testicular tissue	Preclinical (animal studies) [29-31]	Not yet tested for safety and efficacy in humans	Managing expectations; consent for future, unproven technologies

Ethical considerations and psychosocial impact

Informed Consent and Adolescent Autonomy

In pediatric and adolescent oncofertility, ethical tensions frequently arise at the intersection of informed consent, parental decision-making, and emerging child autonomy. Although international bodies recommend that infertility risk and FP options be discussed at diagnosis [5,6,9,18], the capacity of children and adolescents to provide meaningful assent varies with developmental stage, and legal consent typically rests with parents or guardians [6,8]. Prepubertal children often lack the cognitive maturity and future orientation required to comprehend reproductive risk [2,8], whereas adolescents often wish to participate in decisions that may affect their future parenthood, although confidentiality concerns, emotional distress at diagnosis, and limited clinician preparedness may compromise fully informed discussions [2,3].

Adolescents also tend to place greater importance on direct participation in decisions and may be more skeptical of experimental procedures than their parents [32,33]. In a national Australian study of 99 adolescents and young adults and 111 parents, both groups valued adolescent autonomy in fertility decision-making, while many adolescents also appreciated parental support and guidance [34]. FP should therefore be approached as a developmental process rather than a single decision at diagnosis. Young children may require explanations based on concrete concepts and may express preferences through behavior or simple choices, whereas adolescents should receive direct information, opportunities for private conversations with clinicians, and a meaningful role in decision-making.

Parental Decision-Making and Ethical Dilemmas

The ethical principle of beneficence, protecting a child’s potential future fertility, must be balanced against non-maleficence, particularly when procedures such as ovarian or testicular tissue cryopreservation are offered to prepubertal patients with uncertain benefit [4,8,15]. Long-term follow-up of boys who have undergone testicular biopsy underscores both the feasibility of tissue sampling and the uncertainty of ultimate reproductive outcomes, reinforcing the need for transparent counseling [35].

Parents must make these time-sensitive decisions under the stress of a life-threatening diagnosis, balancing beneficence against uncertainty regarding experimental techniques [4,8]. This responsibility can generate guilt, anxiety, and fear of future regret, particularly when proxy decisions may not align with the child’s eventual adult preferences [1,2]. Families may also differ in the importance they place on biological parenthood, acceptable medical risk, and the child’s capacity to participate, while financial strain and cultural or religious beliefs can influence acceptance of FP [11,13,15]. Regulatory and cultural contexts further shape practice, with variable guideline adherence and perceived barriers documented across countries [5,13,15].

Ethical consultation may be especially valuable when parental preferences conflict with the child’s wishes, when procedures remain investigational, or when questions arise concerning stored tissue after death or incapacity. Ethically robust practice therefore integrates parental authority with developmentally appropriate assent, acknowledges the investigational nature of certain interventions, and balances anticipated medical benefit with preservation of future autonomy [6,8].

Psychological Impact on Patients and Families

Fertility discussions occur at a uniquely vulnerable time. Immediately following diagnosis, conversations about future infertility may evoke fear, anxiety, and distress, particularly when invasive procedures are introduced alongside urgent cancer treatment [1,3]. For adolescents, these discussions intersect with emerging body image, sexual identity, and developmental tasks related to autonomy [2,10], and concerns may extend to delayed puberty, sexual functioning, and the possibility that preserved tissue will not result in biological parenthood.

Conversely, receiving clear information and being offered preservation options may promote empowerment, and resilience can emerge when families perceive FP as offering hope for the future, even when procedures remain experimental [1,4]. In a study of 108 parents and 30 patients using the validated Decision Regret Scale, 81.4% of participants reported low regret, which was associated with having a fertility discussion before treatment and with pursuing FP, although some families remained dissatisfied with the decision-making process even when they reported low regret [7]. Counseling should therefore distinguish clearly between the possibility of preserving fertility and the guarantee of future reproductive success, explain fertility risks repeatedly, invite questions, and acknowledge uncertainty.

Role of the Multidisciplinary Team

Healthcare providers influence whether FP is discussed, understood, and accessed. Multidisciplinary counseling involving pediatric oncologists, reproductive specialists, nurses, psychologists, social workers, and ethicists is recommended to support informed, developmentally appropriate decision-making [3,6,8], and early, clear communication before gonadotoxic therapy has been associated with improved satisfaction and reduced regret [1,7]. Communication should be honest about benefits, limitations, experimental status, procedural risks, and costs.

However, barriers persist, including limited provider training, time constraints, inconsistent guideline implementation, and resource disparities across healthcare systems [5,13,15]. Counseling practices may also be shaped by assumptions about age, sex, gender, disability, family structure, or the perceived importance of fertility to a particular child, and younger and female patients have been reported to receive fewer opportunities for specialist consultation [32]. Current guidelines therefore emphasize the role of the oncology team in overcoming barriers to access through advocacy, awareness of bias, and support with financial navigation [18]. Standardized referral pathways, decision aids, interpreters, culturally responsive materials, structured counseling toolkits, and psychological consultation can help reduce these disparities [6,13].

Long-Term Psychosocial Outcomes

Concerns about fertility may become more prominent during puberty, intimate relationship development, and the transition to adulthood. Long-term outcomes may include infertility-related distress, concerns about self-esteem and identity, anxiety about romantic relationships, and uncertainty regarding parenthood, and inadequate counseling at diagnosis may contribute to later dissatisfaction or remorse [1,7]. Nevertheless, many survivors retain a strong desire for children and may view future parenthood as an important component of recovery and adult identity [36]. In a Canadian cohort of 311 long-term survivors, 21.2% reported fertility-related concerns, and among 80 survivors assessed on three occasions, 30% reported concerns at least once and 9% at every assessment [37].

As survivors mature, they may need renewed counseling regarding reproductive function, endocrine health, relationships, disclosure, and available family-building options. Follow-up care should therefore assess psychological well-being alongside gonadal function, with referral for counseling when distress, decisional regret, grief, or relationship difficulties emerge. Integrating multidisciplinary, evidence-informed care throughout survivorship is essential to mitigating long-term distress and fostering resilience [5].

## Conclusions

This review has outlined the fertility risks faced by children and adolescents receiving gonadotoxic treatment, the preservation options available at different developmental stages, and the ethical and psychosocial considerations that shape decision-making. Across these areas, a recurring theme is the uncertainty families face when making consequential decisions on behalf of children, often under considerable time pressure and with incomplete information about future benefit.

We therefore recommend that fertility preservation be approached as an ongoing, developmentally appropriate process rather than a single decision at diagnosis. Early and honest multidisciplinary counseling, standardized referral pathways that promote equitable access, and psychosocial support extending into survivorship should be regarded as central to high-quality care. Future research should prioritize longitudinal studies of how counseling interventions influence the long-term emotional well-being of survivors and their families, so that care protocols evolve alongside advances in reproductive science.
